# Bisphenol A and Adiposity in an Inner-City Birth Cohort

**DOI:** 10.1289/EHP205

**Published:** 2016-05-17

**Authors:** Lori A. Hoepner, Robin M. Whyatt, Elizabeth M. Widen, Abeer Hassoun, Sharon E. Oberfield, Noel T. Mueller, Diurka Diaz, Antonia M. Calafat, Frederica P. Perera, Andrew G. Rundle

**Affiliations:** 1 Department of Environmental Health Sciences, and; 2 Columbia Center for Children’s Environmental Health, Mailman School of Public Health, Columbia University, New York, New York, USA; 3 Department of Environmental and Occupational Health Sciences, School of Public Health, State University of New York Downstate Medical Center, Brooklyn, New York, USA; 4 Department of Epidemiology, Mailman School of Public Health, Columbia University, New York, New York, USA; 5 New York Obesity Nutrition Research Center, Columbia University Medical Center, New York, New York, USA; 6 Institute of Human Nutrition,; 7 Department of Medicine, and; 8 Division of Pediatric Endocrinology, Diabetes and Metabolism, Department of Pediatrics, College of Physicians and Surgeons, Columbia University Medical Center, New York, New York, USA; 9 Department of Epidemiology, Johns Hopkins Bloomberg School of Public Health, Baltimore, Maryland, USA; 10 National Center for Environmental Health, Centers for Disease Control and Prevention, Atlanta, Georgia, USA

## Abstract

**Background:**

Early-life exposure to the endocrine disruptor bisphenol A (BPA) may contribute to the development of obesity. Prospective evidence in humans on this topic is limited.

**Objectives:**

We examined prenatal and early-childhood BPA exposures in relation to childhood measures of adiposity in the Columbia Center for Children’s Environmental Health (CCCEH) New York City birth cohort.

**Methods:**

BPA concentrations were measured in prenatal (n = 375) and child ages 3 (n = 408) and 5 years (n = 518) spot urine samples. Childhood anthropometric and bioelectrical impedance outcomes included body mass index z-scores (BMIZ) at 5 and 7 years, and fat mass index (FMI), percent body fat (%BF), and waist circumference (WC) at 7 years. Associations were evaluated using multiple linear regression with continuous and tertile BPA concentrations.

**Results:**

Prenatal urinary BPA concentrations were positively associated with child age 7 FMI (β = 0.31 kg/m2; 95% CI: 0.01, 0.60, p = 0.04), %BF (β = 0.79; 95% CI: 0.03, 1.55, p = 0.04), and WC (β = 1.29 cm; 95% CI: 0.29, 2.30, p = 0.01), but not BMIZ, or change in BMIZ between ages 5 and 7 years (all p-values > 0.1). FMI results were sex-specific. Child urinary BPA concentrations were not associated with child anthropometric outcomes (all p-values > 0.05).

**Conclusions:**

Analyses of the CCCEH longitudinal birth cohort found associations between prenatal urinary BPA concentrations and FMI, %BF, and WC. Our results suggest that prenatal BPA exposure may contribute to developmental origins of adiposity. These findings are consistent with several prior studies, raising concern about the pervasiveness of BPA.

**Citation:**

Hoepner LA, Whyatt RM, Widen EM, Hassoun A, Oberfield SE, Mueller NT, Diaz D, Calafat AM, Perera FP, Rundle AG. 2016. Bisphenol A and adiposity in an inner-city birth cohort. Environ Health Perspect 124:1644–1650; http://dx.doi.org/10.1289/EHP205

## Introduction

Obesity in children, defined by a body mass index (BMI) greater than or equal to the 95th percentile for age and sex, is an epidemic of great concern in the United States. According to the Robert Wood Johnson Foundation (RWJF), childhood obesity rates (ages 2–19 years) have more than tripled between the years 1980 and 2010 (RWJF 2012). Long-term risks of childhood obesity include metabolic syndrome, type 2 diabetes, cardiovascular disease, and reduced adult life expectancy, with children of minority status at greater risk for becoming obese (Shaibi et al. 2007; Singh et al. 2010; Strauss and Pollack 2001). The 2007–2008 overall prevalence of obesity and overweight was 20.3% and 17.6% for New York City (NYC) public school children, with African American and Hispanic having higher odds than white children of being obese [odds ratio (OR) = 1.11; 95% confidence interval (CI): 1.07, 1.15 and OR = 1.48; 95% CI: 1.43, 1.53, respectively] (Rundle et al. 2012). Early-life exposure to endocrine disruptors, such as bisphenol A (BPA), may be contributing to the obesity epidemic (Grün and Blumberg 2006). Endocrine disruptors may also lead to altered metabolic regulation early in life (Chevalier and Fénichel 2015).

BPA is a known endocrine-disrupting chemical and a key component in polycarbonate plastics and epoxy resins that are commonly used in consumer products, resulting in significant human exposure (Calafat et al. 2008; Vandenberg et al. 2007, 2010). Exposure pathways include oral, dermal, and inhalation, with dietary intake the primary route (Wilson et al. 2003; Zalko et al. 2011). Prenatal exposure may occur *in utero* or by use of catheters during delivery (Ikezuki et al. 2002; Schönfelder et al. 2002; Vandentorren et al. 2011).

Cross-sectional studies suggest that BPA exposure is associated with obesity in children (Bhandari et al. 2013; Eng et al. 2013; Li et al. 2013; Trasande et al. 2012; Wang et al. 2012; Wells et al. 2014). However, because diet is an exposure route, reverse causality cannot be ruled out for these cross-sectional findings because obese children may have greater exposure to BPA due to higher dietary intakes. Longitudinal data on effects of prenatal BPA exposure on postnatal adiposity are limited due to dissimilarities in outcome measures, child age, and geographic geographic location (Braun et al. 2014; Chou et al. 2011; Harley et al. 2013; Lee et al. 2014; Philippat et al. 2014; Snijder et al. 2013; Tang et al. 2013; Valvi et al. 2013). Only two other U.S. studies that investigated BPA exposure and obesity vary in their anthropometric outcomes: body mass index (BMI) trajectories and waist circumference (Braun et al. 2014) and BMI, waist circumference, fat mass, and body fat percent (Harley et al. 2013). We hypothesized that prenatal and early childhood BPA exposure would be positively associated with childhood adiposity at ages 5 and 7 years in a NYC inner-city birth cohort.

## Methods

### Study Design and Population

Study subjects are participants in the prospective birth cohort of the Columbia Center for Children’s Environmental Health (CCCEH) in Northern Manhattan and the South Bronx, New York. Mother–child pairs were selected if a maternal prenatal urine sample was analyzed for BPA (*n* = 375) (see Figure S1). We additionally included children if BPA concentration in urine was measured at child age 3 (*n* = 408) and/or 5 years (*n* = 518) (see Figure S2). The CCCEH study design is described elsewhere (Perera et al. 2003; Whyatt et al. 2003). Briefly, we enrolled 727 women ages 18–35 years old during their third trimester of pregnancy. Women were included if they self-identified as either African American or Dominican and had resided in Northern Manhattan or the South Bronx for at least 1 year before pregnancy. Exclusion criteria included mother’s report of cigarette smoking or use of other tobacco products during pregnancy, illicit drug use, diabetes, hypertension, known HIV, or a first prenatal visit after the 20th week of gestation. Research staff abstracted medical records of the mother and infant at delivery to ascertain prenatal medical history and birth outcomes. We contacted participants every 3 months until child age 5 years and every 6 months thereafter for questionnaire follow-up, with children’s biological samples collected biennially beginning at age 3 years. The CCCEH study has retained documentation regarding loss to follow-up status and reasons for missed intervals.

Data collection was completed through age 7 years in August 2013 and laboratory measurement of urinary BPA was completed for all samples. Study procedures, questionnaires, and collection of biological samples were explained to each subject at enrollment, and a signed consent, approved by the IRB of Columbia University Medical Center and the Centers for Disease Control and Prevention (CDC), was obtained.

### Urinary Biomarker Collection

For mothers, urine was collected between 1999 and 2006 during the third trimester of pregnancy (mean ± SD gestational age, 34 ± 3 weeks) concurrent with administration of the baseline questionnaire by trained bilingual interviewers. In children, urine was collected concurrent with follow-up questionnaires between 2001 and 2010. All samples were spot urine samples collected at varying times of day. The date, but not the exact time of collection, was available.

The total (free plus conjugated) BPA urinary concentrations (ng/mL) were measured as previously described (Calafat et al. 2008). The limit of detection (LOD) was 0.4 ng/mL. Specific gravity, as a measure of urinary dilution, was quantified at room temperature at Columbia University with a handheld refractometer (PAL 10-S; Atago).

We also measured four urinary metabolites of di(2-ethylhexyl) phthalate (DEHP) at CDC: mono(2-ethylhexyl) phthalate, mono(2-ethyl-5-carboxypentyl) phthalate, mono(2-ethyl-5-hydroxyhexyl) phthalate, and mono(2-ethyl-5-oxohexyl) phthalate (Kato et al. 2005; Silva et al. 2007). DEHP metabolite LODs ranged from 0.2 to 1.2 ng/mL. Urinary concentrations of DEHP metabolites and BPA were positively correlated (*r* coefficient range, 0.16–0.34) in the CCCEH birth cohort (Hoepner et al. 2013), and in the Health Outcomes and Measures of the Environment (HOME) study (Braun et al. 2011). BPA was undetectable prenatally (6%) and at child ages 3 years (2%) and 5 years (2%). For the few BPA and DEHP metabolites results < LOD, the value of LOD/2 was substituted, consistent with prior analyses (Whyatt et al. 2003, 2009).

### Anthropometric Measurements

Weight before pregnancy was self-reported by the mother during the third trimester. Birth weight was collected from the hospital medical record. We collected body size measurements and/or body composition data for children age 5 years (*n* = 498) and 7 years (*n* = 511). A Detecto Cardinal 750 digital scale/stadiometer (Cardinal Scale Manufacturing Company) was used to collect standing height measurements at ages 5 and 7 years until January 2010. After January 2010, we assessed standing height with the Holtain-Harpenden Wall Mounted Stadiometer Counter 602VR (Holtain Limited). Body composition values, including fat mass, were collected with the Tanita Digital Body Mass Indicator Scale BC-148 (Tanita Corporation of America) at age 7 years. Children wore light clothing and no shoes. Body mass index *z*-scores (BMIZ) were quantified using the SAS programs provided by the CDC (2011). Fat mass index (FMI) was calculated using the algorithm [fat mass(kg)/height(m)^2^] (VanItallie et al. 1990). Waist circumference (WC) was measured at a level midway between the lower rib margin and iliac crest using a nonstretchable measurement tape.

### Statistical Analysis

BPA concentrations and the molar sum of DEHP metabolite concentrations (ΣDEHP) were natural log (ln)–transformed for analysis to correct for their non-normal distribution. Specific gravity values were standardized by *z*-score transformation to stabilize model constant estimates. The standardized specific gravity concentrations were included in regression models as a covariate except for models with BPA concentration tertiles as the predictor variables. Because using percentiles is a method of ordering by rank, we arithmetically adjusted BPA concentrations at each urine collection interval to calculate tertiles by first accounting for urinary dilution and then ln-transforming using the following formula: Specific gravity adjusted/log-transformed BPA = LN{BPA_raw_ × [(mean specific gravity for population at collection interval – 1)/(individual specific gravity – 1)]} (Hauser et al. 2004). In other words, the prenatal BPA concentration was first specific gravity adjusted and then natural-log transformed.

For childhood BPA exposure, we calculated the mean of BPA concentrations at ages 3 and 5 years. In the absence of two childhood urinary concentrations, the single existing concentration was used (*n* = 51).

Linear regression analyses were conducted to determine whether maternal prenatal urinary BPA concentrations predicted birth weight, BMIZ at ages 5 and 7 years, and the change in BMIZ (ΔBMIZ) from age 5 to 7 years, as well as FMI, percent body fat (%BF), and WC at age 7 years. Additionally, linear regression was used to analyze whether child urinary BPA concentrations from age 3 years or the mean of ages 3 and 5 years predict BMIZ at ages 5 and 7 years, ΔBMIZ from age 5 to 7 years, and FMI, %BF, and WC at age 7 years. To assess the representativeness of the subset analyzed, *t*-tests and chi-square tests were performed to compare subjects with and without prenatal and postnatal BPA concentrations and with and without anthropometric outcomes at 5 years and 7 years of age.

Maternal prenatal urinary BPA concentrations were evaluated separately from child based on prior analyses in the CCCEH birth cohort confirming that prenatal BPA concentrations were not correlated with and were significantly lower than childhood BPA concentrations (Hoepner et al. 2013). However, we separately assessed as potential confounders the prenatal BPA concentrations in childhood predictor regression models and the childhood BPA concentrations in the prenatal predictor regression models and found no confounding. Furthermore, we did not find an association between prenatal BPA concentrations with maternal weight gain during pregnancy, so we did not consider it to be a confounder variable in our analysis.

Variables were tested for in the regression models if we had previously found they were associated with BPA and if they were expected to be associated with dietary patterns and obesity (Hoepner et al. 2013; Rundle et al. 2012). Variables were included in the model if they were significantly (*p* < 0.05) associated with the outcome. The following variables were controlled for in the analyses of child anthropometric measures at ages 5 and 7 years: specific gravity, ln ΣDEHP, race/ethnicity, dichotomous maternal prepregnancy obesity, child sex, birth weight, and gestational age. Models including prenatal BPA concentrations also included prenatal specific gravity and ln ΣDEHP, whereas models including childhood BPA concentrations included childhood specific gravity and ln ΣDEHP. Additionally, we controlled for height in the analyses of outcomes: %BF at age 7 years and WC at age 7 years.

We stratified analyses by child sex in order to explore differences in associations by sex that had been found in other studies (Harley et al. 2013). Possible interactions between BPA concentration and child sex were assessed for all BMIZ and 7-year body composition outcomes.

We conducted sensitivity analyses using baseline data and logistic regression models to estimate inverse probability weighting (IPW) for successful follow-up to assess potential bias of effect estimates due to loss to follow-up and missing anthropometric data (Curtis et al. 2007; Hernán et al. 2004; Robins et al. 2000; Rundle et al. 2012; Widen et al. 2015). To estimate the weights, we included all variables in each of our final models, mother’s satisfaction with living conditions, mother’s years of school completed at time of pregnancy, as well as geographic information system variables using 2000 U.S. Census block–group data aggregated to 1-km radial neighborhood buffers around the home: linguistic isolation and neighborhood socioeconomic status (Rundle et al. 2012).

For all analyses, we considered results with *p* < 0.05 to be statistically significant. Analyses were performed using IBM SPSS Statistics version 22.0 (IBM Corp.) and Stata version 13.0 (StataCorp).

## Results

Demographic and clinical characteristics of the 369 participants with prenatal BPA concentrations and available birth outcomes data are presented in Table 1. Table 2 shows the characteristics of the study participants grouped by BPA concentration interval and age at which anthropometric measures were collected. There was no significant difference between subjects with and without prenatal and postnatal BPA concentrations. Additionally, there was no significant difference between subjects with and without anthropometric outcomes at 5 years and 7 years of age.

**Table 1:** Characteristics of mothers and children with available birth weight and/or prenatal weight gain, and prenatal urinary BPA measures (*n* = 369).

Characteristic	Value
Categorical variables [*n* (%)]
Sex of child
Female	201 (54.5)
Male	168 (45.5)
Race/ethnicity
African-American	131 (35.5)
Dominican	238 (64.5)
Foreign born^*a*^
USA born	162 (44)
Foreign born	206 (56)
Maternal prepregnancy obesity (BMI > 30 kg/m^2^)^*a*^
Yes	75 (20.3)
No	285 (77.2)
Parity
Nulliparous	168 (45)
Multiparous	201 (54)
Continuous variables (mean ± SD)
Birth weight (g)	3,365 ± 475
Maternal prepregnancy BMI^*a*^ (kg/m^2^)	25.80 ± 6.00
Prenatal BPA (ng/mL)	3.03 ± 4.16
^***a***^Subjects missing data on each variable: Foreign born *n* = 1, Prepregnancy obese *n* = 9, Prepregnancy BMI *n* = 9.	

**Table 2:** Characteristics of subjects with urinary BPA measures and childhood anthropometric outcomes.

Characteristic	Prenatal BPA		Age 3 years BPA	Age 3 and/or 5 years BPA
	5 year anthropometry (*n* = 300)	7 year anthropometry (*n* = 308)	5 year anthropometry (*n* = 317)	7 year anthropometry (*n* = 325)
Categorical variables [*n* (%)]
Sex of child
Female	165 (55)	164 (53)	164 (52)	173 (53)
Male	135 (45)	144 (47)	153 (48)	152 (47)
Race/ethnicity
African American	113 (38)	113 (37)	131 (41)	136 (42)
Dominican	187 (62)	195 (63)	186 (59)	189 (58)
Prepregnancy obesity (BMI ≥ 30 kg/m^2^)
Yes	64 (21)	65 (21)	71 (22)	71 (22)
Child overweight/obese status
Overweight (BMI 85th–95th percentile)	49 (16)	64 (21)	52 (16)	58 (18)
Obesity (BMI ≥ 95th percentile)	54 (18)	72 (23)	67 (21)	86 (26)
Continuous variables (mean ± SD)
Prepregnancy weight (g)	67.24 ± 17.64	67.58 ± 17.69	68.35 ± 18.16	68.78 ± 18.12
Prepregnancy BMI	25.66 ± 6.11	25.79 ± 6.14	25.87 ± 6.11	25.95 ± 6.13
Birth weight (g)	3,356 ± 480	3,371 ± 487	3,381 ± 492	3,385 ± 499
BMI	16.52 ± 2.73	17.93 ± 3.43	16.78 ± 2.82	18.19 ± 3.62
BMI *z*-score	0.42 ± 1.42	0.79 ± 1.15	0.58 ± 1.37	0.86 ± 1.15
Median BMI percentile (interquartile range)	69.47 (32.36–92.13)	81.12 (53.38–94.27)	74.88 (37.40–93.11)	81.17 (54.24–95.72)
Fat mass (kg)	—	7.19 ± 3.92	—	7.47 ± 4.09
FMI^*a*^	—	4.51 ± 2.18	—	4.67 ± 2.25
Percent body fat^*a*^	—	24.13 ± 6.06	—	24.53 ± 6.14
Waist circumference^*a*^ (cm)	—	23.50 ± 8.27	—	23.50 ± 8.43
Prenatal BPA (ng/mL)	3.12 ± 4.42	3.06 ± 4.35	—	—
Child BPA (ng/mL)	—	—	8.12 ± 12.70	3 year: 8.05 ± 12.53 5 year: 5.35 ± 6.51 3 and/or 5 year: 6.72 ± 7.43
^***a***^Subjects with prenatal BPA concentration and missing data on each variable at age 7 years: fat mass index *n* = 8, fat mass *n* = 8, percent body fat *n* = 8, waist circumference *n* = 11; subjects with mean childhood BPA concentration and missing data on each variable at age 7 years: fat mass index *n* = 22, fat mass *n* = 22, percent body fat *n* = 22, waist circumference *n* = 28.				

### Prenatal BPA Concentration versus Child Anthropometry

Prenatal BPA concentrations were not associated with birth weight (see Table S1). Results from analyses of fully adjusted ln-transformed prenatal BPA concentrations and child anthropometric outcomes are shown in Table 3. Overall, prenatal BPA concentrations were not significantly associated with BMIZ at ages 5 years or 7 years or ΔBMIZ from 5 to 7 years. Prenatal BPA concentrations were positively associated with FMI at age 7 years (β = 0.31 kg/m^2^; 95% CI: 0.01, 0.60, *p* = 0.04,). Prenatal BPA concentrations were also positively associated with %BF at age 7 years (β = 0.79, *p* = 0.04) and with WC at age 7 years (β = 1.29 cm, *p* = 0.01).

**Table 3:** Associations between prenatal urinary BPA concentrations and child anthropometric outcomes [β coefficient (95% CI)].

BPA measures	Age 5 years	Change from age 5 to 7 years	Age 7 years			
	BMI *z*-score	BMI *z*-score	BMI *z*-score	FMI	Percent body fat	Waist circumference (cm)
Continuous ln-transformed BPA concentrations^*a*^^,^^*b*^
Prenatal BPA	(*n* = 300) 0.04 (–0.16, 0.24)	(*n* = 279) 0.06 (–0.06, 0.18)	(*n* = 308) 0.11 (–0.04, 0.26)	(*n* = 300) 0.31* (0.01, 0.60)	(*n* = 300) 0.79* (0.03, 1.55)	(*n* = 297) 1.29* (0.29, 2.30)
Tertiles of specific gravity-adjusted ln-transformed prenatal BPA concentrations (in ng/mL)^*b*^^,^^*c*^
< 0.33	Reference	Reference	Reference	Reference	Reference	Reference
0.33–0.98	–0.29 (–0.70, 0.12)	0.16 (–0.09, 0.40)	–0.10 (–0.42, 0.21)	0.004 (–0.61, 0.40)	0.13 (–1.45, 1.71)	0.89 (–1.21, 3.00)
> 0.98	–0.09 (–0.51, 0.32)	0.16 (–0.09, 0.41)	0.10 (–0.22, 0.42)	0.47 (–0.14, 1.09)	0.73 (–0.86, 2.32)	1.93 (–0.20, 4.06)
^***a***^All analyses controlled for maternal variables: prepregnancy obesity, race/ethnicity, prenatal ∑DEHP, prenatal urinary specific gravity; child variables: sex, birth weight, gestational age.^***b***^Additionally, height was controlled for in analyses of percent body fat and waist circumference. ^***c***^All analyses controlled for maternal variables: prepregnancy BMI, race/ethnicity, prenatal ∑DEHP; child variables: sex, birth weight, gestational age. *p < 0.05. #*p* < 0.1.						

To determine whether the FMI results were attributable to the fat mass or the height component of the index, we substituted the fat mass itself as the dependent variable in the model. Prenatal BPA concentrations were positively associated with fat mass regardless of the addition of height as a covariate (adjusting for height: β = 0.55 kg, *p* = 0.02; without adjustment for height: β = 0.57 kg, *p* = 0.03). Prenatal BPA concentrations and height were not associated. Exclusion of the prenatal ln ΣDEHP covariate from the model not appreciably change the associations.

### Prenatal Tertile Analysis

Linear regression analysis using tertiles of prenatal BPA concentrations as predictor variables, with the first tertile as the reference, suggested positive linear associations with FMI, %BF, and WC (Table 3).

### Prenatal Differences by Child Sex

After stratifying by sex, among girls there was a positive association between prenatal BPA concentrations and 7-year FMI and WC (Table 4). Among boys, there was no association between prenatal BPA concentrations and anthropometric outcomes. The interaction between sex and BPA concentrations was significant (Table 4).

**Table 4:** Associations*^a^* between ln-transformed prenatal urinary BPA concentrations and child anthropometric outcomes stratified by sex and age 7 year interaction analysis [β coefficient (95% CI)].

Prenatal BPA (ng/mL)	Age 5 years	Change from age 5 to 7 years	Age 7 years			
	BMI *z*-score	BMI *z*-score	BMI *z*-score	FMI	Percent body fat	Waist circumference (cm)
Girls	(*n* = 165) 0.02 (–0.26, 0.30)	(*n* = 153) 0.04 (–0.14, 0.20)	(*n* = 164) 0.12 (–0.10, 0.33)	(*n* = 161) 0.48* (0.5, 0.91)	(*n* = 161) 0.74 (–0.32, 1.81)	(*n* = 160) 1.45* (0.5, 2.85)
Boys	(*n* = 135) 0.09 (–0.20, 0.37)	(*n* = 126) 0.03 (–0.14, 0.21)	(*n* = 144) 0.10 (–0.12, 0.32)	(*n* = 139) 0.06 (–0.35, 0.47)	(*n* = 139) 0.73 (–0.40, 1.87)	(*n* = 137) 1.07 (–0.47, 2.61)
*p*-Value (BPA) × (sex)	—	—	0.41	0.04*	0.51	0.32
^***a***^All analyses controlled for maternal variables: prepregnancy obesity, race/ethnicity, prenatal ∑DEHP, prenatal urinary specific gravity; child variables: birth weight, gestational age. Additionally, height was controlled for in analyses of percent body fat and waist circumference. *p < 0.05. #*p* < 0.1						

### Postnatal BPA Concentration versus Child Anthropometry

Child BPA concentrations had a borderline negative association with ΔBMIZ from 5 to 7 years (β = –0.10; 95% CI: –0.02, 0.005, *p* = 0.06). Child BPA concentrations were not associated with FMI, %BF, or WC at age 7 years (see Table S2).

### Postnatal Tertile Analysis

Linear regression analysis using tertiles of child BPA concentrations as predictor variables, with the first tertile as the reference, was consistent with the borderline negative association with ΔBMIZ from 5 to 7 years (see Table S2). Higher exposure to BPA may be associated with lower ΔBMIZ.

### Postnatal Differences by Child Sex

When we conducted analyses stratified by sex we found a negative association between child urinary BPA concentrations and ΔBMIZ from 5 to 7 years for girls and no associations for boys (see Table S3). Interaction terms for postnatal BPA concentrations and sex were not significant.

### IPW Analysis

Weighting the data by the inverse probability of follow-up and complete anthropometric and biomarker data collection by 7 years did not appreciably change the size of the effect estimates (data not shown).

## Discussion

We observed a positive association between prenatal urinary BPA concentration and childhood FMI, %BF, and WC at age 7 years. These results suggest prenatal BPA exposure is associated with overall body fat and central adiposity, accounting for height. However, contrary to our hypotheses, we found that maternal urinary BPA concentrations were not associated with birth weight, childhood BMIZ at ages 5 and 7 years, and ΔBMIZ from age 5 to 7 years. The differences in results across anthropometric outcomes may reflect differences in the construct validity of body composition versus BMI as measures of child adiposity. BMIZ alone may not be the best measure of adiposity in prepubertal children (Mueller et al. 2013). The few pediatric studies in the United States that evaluated BMI versus FMI concluded that these measures are not equivalent for determining excess adiposity in children (Weber et al. 2013). This is particularly important when considering sex because the body composition components of fat mass and lean body mass differ between sexes (Weber et al. 2013). Literature on birth cohort studies of childhood obesity outcomes related to BPA exposure is limited, and only one study included longitudinal analyses of prenatal BPA exposure effects on school-age boys and girls (Braun et al. 2014; Harley et al. 2013; Philippat et al. 2014; Valvi et al. 2013).

In our analysis, we found sex-specific associations with prenatal BPA exposures and FMI at age 7 years. In the Cincinnati, Ohio–based HOME study, a prospective cohort composed primarily of white (67%) and African-American (27%) children (*n* = 297), prenatal urinary BPA concentrations were not associated with BMI at ages 2–5 years (Braun et al. 2014). In the aforementioned study, an increase in childhood BMIZ slope per prenatal BPA tertile was observed, but sex-specific associations were weak and based on a small sample size (Braun et al. 2014). However, accelerated growth may be attributable to a variety of factors including fat mass and fat-free mass that can be determined only with body composition techniques.

Conversely, the “INfancia y Medio Ambiente” (INMA) population-based birth cohort in Spain reported positive associations between prenatal urinary BPA concentration and body size outcomes BMI and WC at age 4 years and no sex differences (Valvi et al. 2013). Others did not identify an association of prenatal urinary BPA with weight or WC at age 3 years in a French birth cohort subset of boys only (Philippat et al. 2014). In the Center for the Health Assessment of Mothers and Children of Salinas (CHAMACOS) birth cohort consisting primarily (98%) of Mexican-American mothers and children in California, researchers found prenatal urinary BPA concentrations to be inversely related to 9 year BMIZ and %BF for girls only; concurrent urinary BPA concentrations were positively related to BMI, fat mass, and WC for both sexes at age 9 years (Harley et al. 2013). Similar to our results, early childhood (5 years) urinary BPA concentrations in the CHAMACOS cohort were not related to late childhood (9 years) anthropometric outcomes. In addition, *in vitro* and *in vivo* experimental studies have shown positive associations between BPA exposure and adipogenesis (García-Arevalo et al. 2014; Masuno et al. 2002; Riu et al. 2011; Rubin et al. 2001; Somm et al. 2009). Our results are an important addition to the growing literature on the potential role of BPA in the developmental origin of overweight, obese, and adipose deposition.

The longitudinal cohort design of this study is a major strength because prior cross-sectional studies (Bhandari et al. 2013; Li et al. 2013; Trasande et al. 2012; Wang et al. 2012) may be biased due to associations between body size and higher food intake, leading to higher exposures to BPA from food. Strengths of our study include collecting repeated urinary BPA concentrations and direct assessment of childhood anthropometric outcomes, with the addition of bioelectrical impedance body composition measures at age 7 years. The analyses found consistent results across the outcomes of FMI, %BF, and WC, although these are inter-correlated.

Another strength of our study is our ability to control for socioeconomic and additional environmental factors, including urinary phthalate concentrations measured concurrently with BPA. Additionally, our study design provides the opportunity for analysis of adiposity in understudied inner-city minority populations. Although obesity prevalence among Hispanic children increased by 24.2% from 2003 to 2007 (Singh et al. 2010), there is little prior information on prenatal and early childhood exposure to BPA and effects on body size outcomes among U.S. minority populations. Although obesity prevalence among African-American children increased 1.75% from 2003 to 2007, according to National Health and Nutrition Examination Survey (NHANES) data from 1999 to 2012, Hispanic and African-American children had an obesity prevalence of 20.9% and 20.3%, respectively (Skinner and Skelton 2014). Furthermore, although the NHANES reports on BPA concentrations among different ethnic groups, it is limited to adults and children ≥ 6 years of age (Calafat et al. 2008). To date, no other study has evaluated the potential BPA effects on childhood body size in a birth cohort composed entirely of more than one minority group. According to the 2010 U.S. Census, African Americans and Hispanics are the dominant minority populations in NYC (25.5% and 28.6%, respectively) (U.S. Census Bureau 2012). In our NYC-based birth cohort, composed of African-American and Dominican mother–child dyads currently being followed through adolescence, we found associations between prenatal BPA exposures and age 7 years anthropometric outcomes measures.

The use of spot urine samples is a potential limitation of our study. Prior studies of minority populations in the United States found a low intraclass correlation (ICC) for BPA between serial urine samples from pregnant women in Puerto Rico (ICC = 0.27) (Meeker et al. 2013) and African-American and Hispanic children in NYC (ICC = 0.22) (Teitelbaum et al. 2008). If, as expected, the exposure misclassification because of variability of urinary BPA concentrations is nondifferential in regard to the outcome, bias toward the null is expected. Thus, due to the poor reliability of the biomarkers, the findings for FMI, %BF, and WC are likely to be underestimates of the true effects.

Although our study was limited by the lack of dietary data during pregnancy and childhood, accurate dietary data are extremely difficult to acquire from young children given age-related development of language skills and recall ability. Dietary measures would also require quantification of BPA in food items, which was outside the scope of this study. Another possible limitation is that body composition at age 7 years was assessed with bioelectrical impedance analysis, which has been validated in some populations, but has not been compared to gold-standard reference methods in a population similar to ours (Haroun et al. 2009). Also, sex differences in pubertal development may be associated with body composition (Ahmed et al. 1999; Blum et al. 1997; Kaplowitz 2008). Earlier puberty in girls may lead to an estrogen-mediated increase in body fat (Kaplowitz 2008). Hormonal and precocious puberty data were not available for this cohort. Therefore, pubertal timing cannot be ruled out as a possible driver of the sex-specific association observed between prenatal BPA concentrations and 7 year FMI and WC. Finally, although we had missing anthropometric and biomarker data from the children in our cohort, IPW analysis suggested that loss to follow-up did not bias our results.

## Conclusions

In our longitudinal birth cohort, we found positive associations between prenatal urinary BPA concentrations and adiposity measures at age 7 years: FMI, %BF, and WC. The association with FMI was sex-specific for girls. Thus, future studies of environmental effects on childhood adiposity may be guided to include puberty assessment. Our findings suggest that prenatal BPA exposure may have an effect on adiposity as children age, an effect that cannot be observed by BMI-based measures alone. Our study was the first to examine and show associations between prenatal exposure to BPA and adiposity outcomes in school-aged children. As the CCCEH cohort ages into adolescence and emerging adulthood, follow-up studies will be critical for evaluating whether the association between prenatal BPA and adiposity persists over time.

## Supplemental Material

(177 KB) PDF
