# Supplementary material for: Bisphenol A and Adiposity in an Inner-City Birth Cohort
Source: Environ Health Perspect. 2016 May 17;124(10):1644–50. doi: 10.1289/EHP205 (PMC5047776; doi:10.1289/EHP205)
Supplement: (177 KB) PDF [file EHP205.s001.acco.pdf]

Environ Health Perspect
DOI: 10.1289/EHP205

Note to readers with disabilities: *EHP* strives to ensure that all journal content is accessible to all readers. However, some figures and Supplemental Material published in *EHP* articles may not conform to 508 standards due to the complexity of the information being presented. If you need assistance accessing journal content, please contact ehp508@niehs.nih.gov. Our staff will work with you to assess and meet your accessibility needs within 3 working days.

Supplemental Material

Bisphenol A and Adiposity in an Inner-City Birth Cohort

Lori A. Hoepner, Robin M. Whyatt, Elizabeth M. Widen, Abeer Hassoun, Sharon E. Oberfield, Noel T. Mueller, Diurka Diaz, Antonia M. Calafat, Frederica P. Perera, and Andrew G. Rundle

Table of Contents

Figure S1. Flowchart of enrollment for prenatal urinary BPA concentrations and childhood anthropometric outcomes

Figure S2. Flowchart of enrollment for childhood urinary BPA concentrations and childhood anthropometric outcomes

Table S1. Associations between prenatal urinary BPA concentrations and birth outcomes

Table S2. Associations between child urinary BPA concentrations and child anthropometric outcomes

Table S3. Associations between child urinary BPA concentrations and child anthropometric outcomes stratified by sex

Figure S1. Flowchart of enrollment for prenatal urinary BPA concentrations and childhood anthropometric outcomes

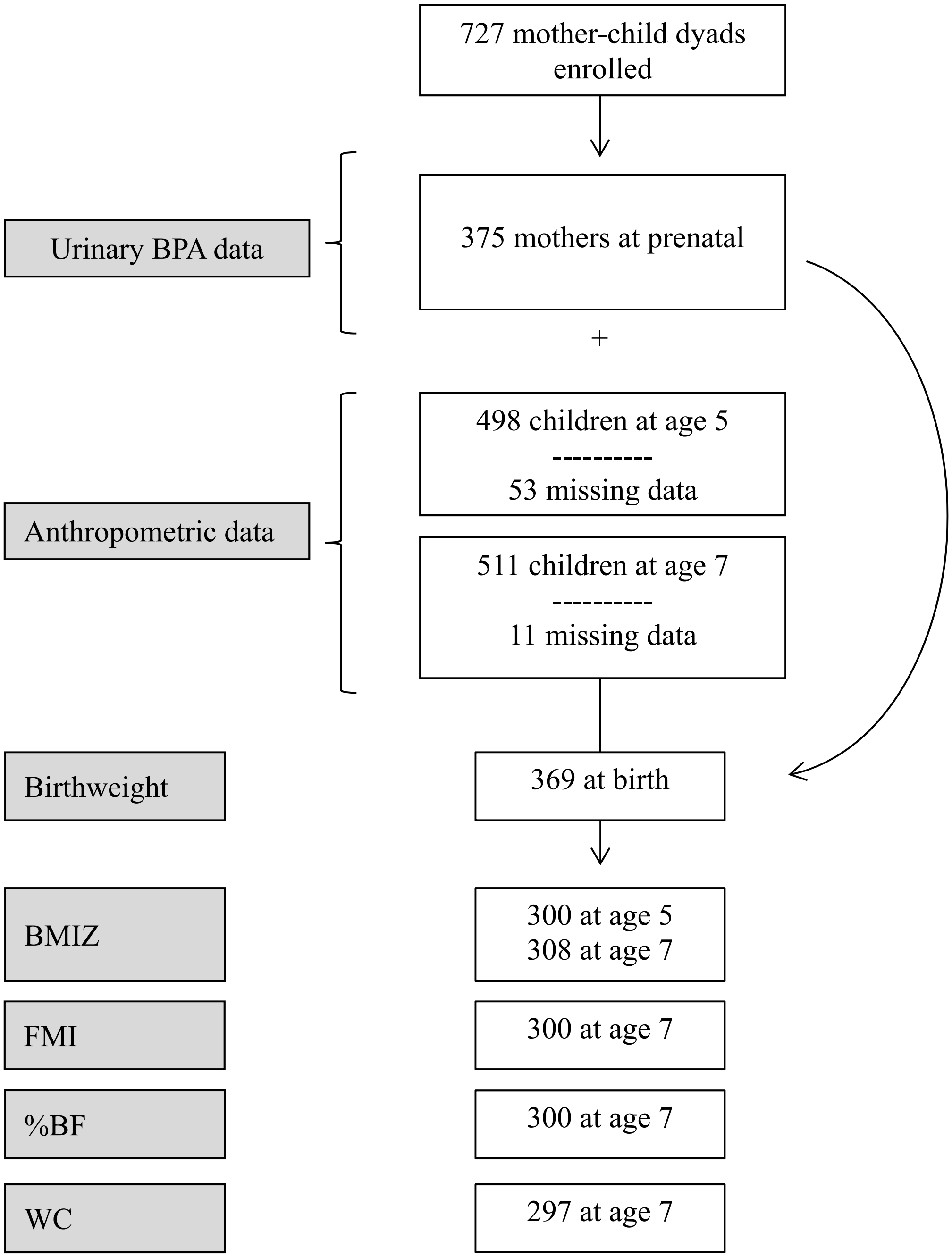

Figure S2. Flowchart of enrollment for childhood urinary BPA concentrations and childhood anthropometric outcomes

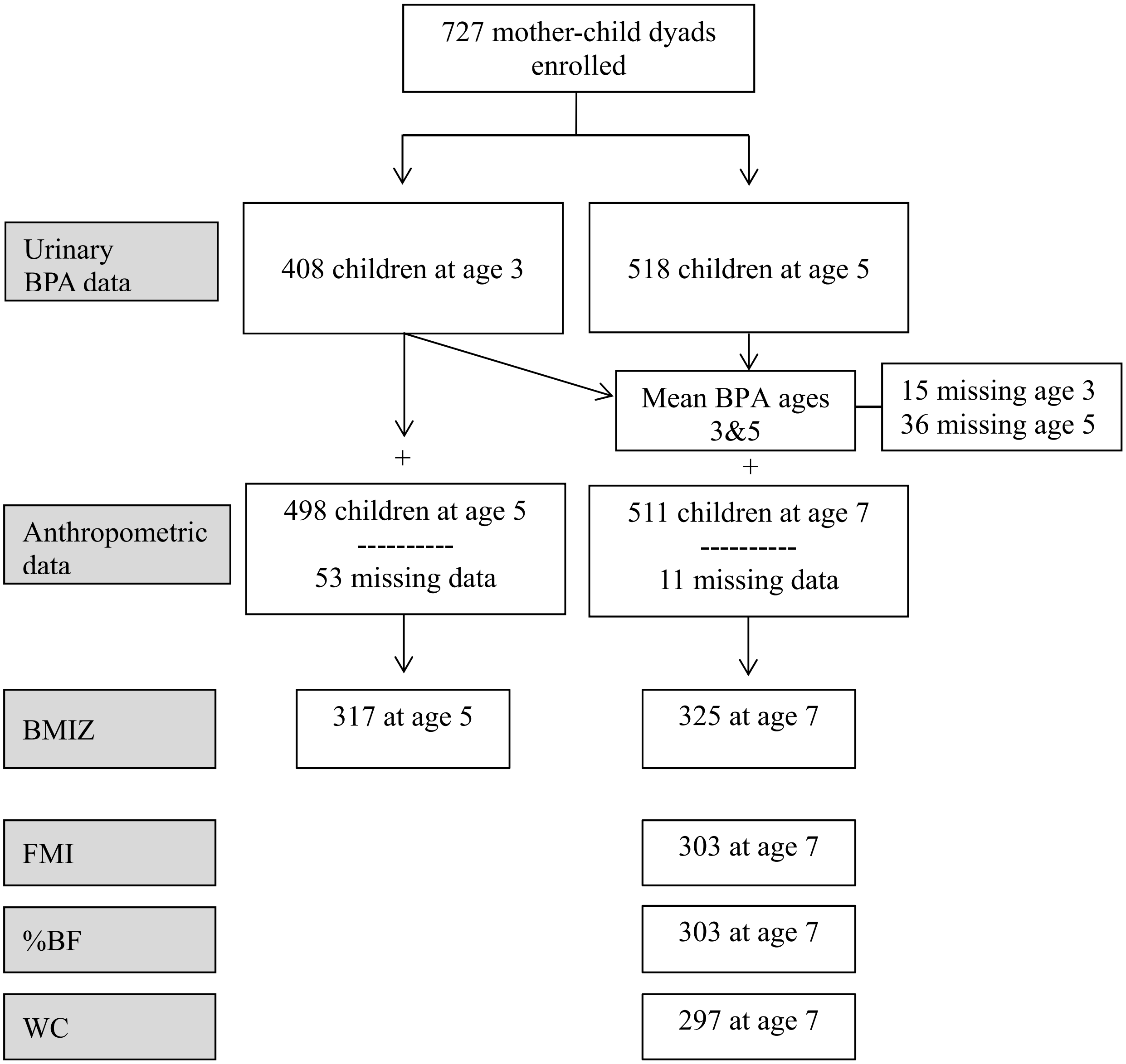

Table S1. Associations between prenatal urinary BPA concentrations and birth outcomes

BPA measures	Birthweight (g)<br>Beta Coefficient (95% CI)
Continuous ln-transformed BPA concentrations	
Prenatal BPA	(N=333)<br>-47.10<br>(-103.70, 9.50)
Tertiles of specific gravity-adjusted ln-transformed prenatal BPA concentrations (in ng/mL)	
<0.33	Reference
0.33-0.98	-85.91<br>(-204.70, 32.88)
>0.98	-79.04<br>(-197.35, 39.26)

The following variables were controlled for in the analysis of continuous ln-transformed BPA concentrations and birth weight: standardized prenatal specific gravity, prenatal ∑DEHP, race/ethnicity, maternal pre-pregnancy BMI, baby sex, maternal foreign born, gestational age, maternal pregnancy weight gain. The following variables were controlled for in the analysis of tertiles of BPA concentrations and birth weight: prenatal ∑DEHP, race/ethnicity, maternal pre-pregnancy BMI, baby sex, maternal foreign born, gestational age, maternal pregnancy weight gain.

After full adjustment in the linear regression model, ln-transformed prenatal BPA concentrations were not associated with birth weight. Linear regression analysis using tertiles of BPA as predictor variables support the lack of association between prenatal urinary BPA concentration and birthweight.

Table S2. Associations between child urinary BPA concentrations and child anthropometric outcomes

	Beta Coefficient (95% CI)					
	Age 5 Yrs	Change From Age 5 to 7 Yrs	Age 7 Yrs			
BPA measures	BMI Z-score			FMI	Percent Body Fat	Waist Circumference (cm)
Continuous ln-transformed BPA concentrations[a,c]						
3 year BPA	(N=317) 0.03 (-0.14, 0.19)					
Tertiles of specific gravity-adjusted ln-transformed 3 year BPA concentrations (in ng/mL)[2]						
<0.98	Reference					
0.98-1.73	-0.13 (-0.50, 0.24)					
>1.73	0.13 (-0.27, 0.48)					
Continuous ln-transformed BPA concentrations[a,c]						
Mean BPA (3-5 years)		(N=298) -0.10[#] (-0.20, 0.004) p=0.06	(N=325) -0.01 (-0.15, 0.14)	(N=303) 0.06 (-0.23, 0.35)	(N=303) 0.17 (-0.58, 0.92)	(N=297) -0.02 (-1.01, 0.96)
Tertiles of specific gravity-adjusted ln-transformed mean BPA concentrations (in ng/mL)[b]						
<1.05		Reference	Reference	Reference	Reference	Reference
1.05-1.78		-0.02 (-0.24, 0.20)	0.21 (-0.10, 0.52)	0.33 (-0.30, 0.96)	0.52 (-1.10, 2.15)	-0.41 (-2.55, 1.73)
>1.78		-0.18 (-0.41, 0.05)	0.01 (-0.31, 0.33)	0.12 (-0.53, 0.77)	0.21 (-1.45, 1.88)	-0.53 (-2.72, 1.66)

[a] All analyses controlled for: maternal variables: pre-pregnancy BMI, race/ethnicity; child variables: sex, birth weight, gestational age, child ∑DEHP, child urinary specific gravity.
[b] All analyses controlled for: maternal variables: pre-pregnancy BMI, race/ethnicity; child variables: sex, birth weight, gestational age, child ∑DEHP.
[c] Additionally, height was controlled for in analyses of percent body fat and waist circumference.
#$p<0.1$

Table S3. Associations[a] between ln-transformed child urinary BPA concentrations and child anthropometric outcomes stratified by sex

	Beta Coefficient (95% CI)					
	Age 5 Yrs	Change From Age 5 to 7 Yrs	Age 7 Yrs			
BPA measures (ng/mL)	BMI Z-score			FMI	Percent Body Fat	Waist Circumference (cm)
Girls						
3 year BPA	(N=164) 0.05 (-0.18, 0.28)					
Mean BPA (3-5 years)		(N=161) -0.18* (-0.32, -0.04) p=0.01	(N=173) -0.13 (-0.33, 0.07)	(N=163) -0.15 (-0.52, 0.23)	(N=163) -0.13 (-1.09, 0.84)	(N=160) -0.15 (-1.50, 1.20)
Boys						
3 year BPA	(N=153) 0.01 (-0.22, 0.25)					
Mean BPA (3-5 years)		(N=137) -0.02 (-0.18, 0.14)	(N=152) 0.12 (-0.10, 0.34)	(N=140) 0.23 (-0.24, 0.69)	(N=140) 0.48 (-0.76, 1.71)	(N=137) 0.06 (-1.46, 1.57)

[a] All analyses adjusted for: maternal variables: pre-pregnancy BMI, race/ethnicity; child variables: birth weight, gestational age, child ∑DEHP, child urinary specific gravity. Additionally, height was controlled for in analyses of percent body fat and waist circumference.

*p < 0.05 #p<0.1
